# Identification of Two Novel Immune Subtypes Characterized by Distinct Prognosis and Tumor Microenvironment in Osteosarcoma

**DOI:** 10.1155/2022/2181525

**Published:** 2022-10-08

**Authors:** Shunhan Yao, Meiling Deng, Xiaojing Du, Qingfeng Chen, Rongzhi Huang

**Affiliations:** ^1^Medical College, Guangxi University, Nanning, Guangxi 530000, China; ^2^School of Information and Management, Guangxi Medical University, Nanning, Guangxi 530021, China; ^3^School of Computer, Electronic and Information, Guangxi University, Nanning, Guangxi 530000, China; ^4^Department of Computer Science and Information Technology, La Trobe University, Melbourne, Victoria 3086, Australia; ^5^Traumatic Orthopedic Hand Surgery, The First People's Hospital of Qinzhou, Qinzhou, Guangxi 535000, China

## Abstract

Osteosarcoma is a kind of primary malignant tumor of bone. In recent years, its therapeutic effect and prognostic survival are dissatisfactory. The tumor immune microenvironment (TIME) reflects immune status of patients, but it is little known in osteosarcoma. Therefore, this study attempts to conduct a comprehensive analysis to explore TIME of osteosarcoma and identify TIME-related subtypes for clinical management and treatment. We successfully established two novel tumor immune infiltration clusters (TIIC) which are characterized by difference of microenvironment and immune-related biological processes. High tumor immune infiltration cluster (H-TIIC) subtypes with higher immune infiltration score shows a better overall survival. Further, the two immune subtypes are shown to differ in immunotherapy and chemotherapy. The results would be helpful for clinical decision in osteosarcoma.

## 1. Introduction

Osteosarcoma is a primary bone malignancy which is considered to be second leading cause of tumor-related death in adolescents aged 10 to 20 years [1, 2]. The five-year survival rate has been obviously improved with application of neoadjuvant chemotherapy [3]. However, in recent years, more and more patients have gradually increased their resistance to chemotherapy. The therapeutic effect and prognostic survival are not satisfactory [4]. The therapeutic scheme and prognosis estimation depend on TNM staging and Huvos Grading, but there are obvious limitations [5]. Thus, it is necessary to establish a new standard to guide an individualized administration of osteosarcoma.

Researches revealed that immune microenvironment is closely associated with tumor. CD8+ T cells could recognize tumor-associated antigens and launch an immune attack against the tumors [6]. Antigen-presenting cells (APCs) such as natural killer (NK) cells, macrophage, and dendritic cells (DC) could express immune checkpoint molecules which inhibited the antitumor immune function of T cells [7]. Tumor-associated macrophages (TAM) are closely associated with tumor metastasis and poor prognosis in osteosarcoma [8]. These evidences indicated that difference of individual immune is an important factor in chemotherapy resistance and prognosis. Therefore, the identification of new immune subtypes based on the immune microenvironment is beneficial to the individualized administration of osteosarcoma.

Tumor immune microenvironment (TIME) represents a complex interactions of tumor-infiltrating immune cell populations and individual immune status [9]. Previous studies have revealed that it could be accurately quantified by single-sample gene set enrichment analysis (ssGSEA) algorithm based on immune gene sets [10–13]. However, the exploration of TIME in osteosarcoma has rarely been reported.

Therefore, ssGSEA algorithm was conducted to explore the TIME and further improve the understanding of immune status of osteosarcoma in this study. Then, nonnegative matrix factorization (NMF) consensus clustering analysis was used to identify two novel immune subtypes in osteosarcoma. At the same time, in order to facilitate clinical application, a simplified model was constructed to distinguish the immune subtypes through sophisticated machine learning algorithms. This study would provide useful help for clinical management and treatment in osteosarcoma.

## 2. Materials and Methods

### 2.1. Data Processing

Firstly, RNA-seq raw data of osteosarcoma were acquired from the Therapeutically Applicable Research to Generate Effective Treatments (TARGET) database (https://ocg. http://cancer.gov/programs/target) through TCGA biolinks R package. And its corresponding clinical data were obtained from UCSC Xena (https://xenabrowser.net). Subsequently, Ensembl IDs were converted to gene symbols based on Ensembl database (http://uswest.ensembl.org/index.html). Raw data were normalized based on variance stabilizing transformation through the DESeq2 R package. Next, GSE21257 of osteosarcoma data [14] was downloaded by GEOquery R package. Probe IDs were converted to gene symbols based on GPL10295 file. The GSE21257 was normalized by limma R package. Finally, samples without follow-up data and gene expression in more than half of the samples with 0 were excluded.

### 2.2. Identification of Immune Subtypes

We quantified TIME level of each sample with 28 immune signatures (S1) based on the ssGSEA algorithm of GSVA R package [10, 15]. Subsequently, it was normalized by using an equation. Next, we used the NMF R package to construct immune subtypes [16]. For the NMF method, “nsNMF” option was selected and 50 iterations were carried out, and the number of clusters *k* was set as 2 to 6. Finally, immune infiltration score of each osteosarcoma sample conducted by the ESTIMATE algorithm and was applied to identify immune subtypes [17].

### 2.3. Survival Analysis

The Kaplan-Meier curves and log-rank test were conducted to confirm the survival differences between two immune subtypes by using survminer R package.

### 2.4. Gene Set Enrichment Analysis

We used DESeq2 R package to conduct a gene list in TCGA cohort. The genes with false discovery rate (FDR) less than 0.05 were performed gene set enrichment analysis by using the fgsea R package. The biological processes with a *P* value less than 0.05 and an absolute value of the normalized enrichment score (NES) more than 2.5 were considered statistically significant.

### 2.5. Immune Response Prediction

The subclass mapping approach [18] was used to predict the clinical response of osteosarcoma immune subtypes to immune checkpoint blockade in TARGET cohort. The same method was also adopted to verify the results in GSE21257 cohort.

### 2.6. Exploration of Sensitivity of Chemotherapy

The pRRophetic R package was used to calculate sensitivity of chemotherapy of each osteosarcoma based on the largest publicly available pharmacogenomics database (Genomics of Drug Sensitivity in Cancer (GDSC), https://www.cancerrxgene.org/) [19]. Three commonly used drugs were selected: cisplatin, methotrexate, and doxorubicin. All parameters were set as the default values. Differences in sensitivity of chemotherapy were determined by the Wilcoxon rank-sum test.

### 2.7. Statistical Analysis

Statistical analysis was based on R4.0 and its corresponding software packages. Continuous and categorical clinical variables between two subtypes were compared using the Wilcoxon rank-sum test and chi-square test. Differences of immune cell infiltration were tested through the Wilcoxon rank-sum test. The association between immune checkpoint proteins and immune subtypes was determined by *t*-test. In all analyses, *P* values < 0.05 indicated statistically significant difference.

## 3. Results

### 3.1. Identification of Two Novel Immune Subtypes

**Figure 1:**
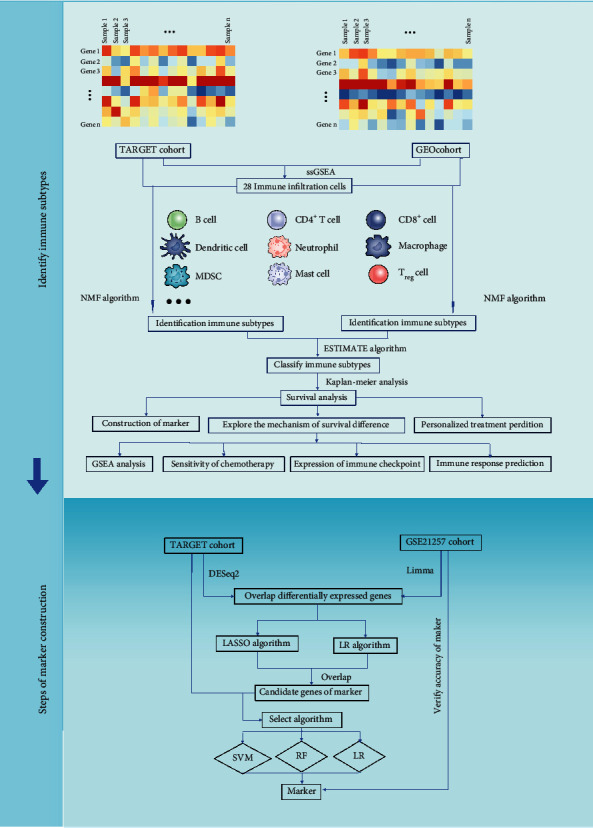
Flowchart of the analyses in this study.

**Figure 2:**
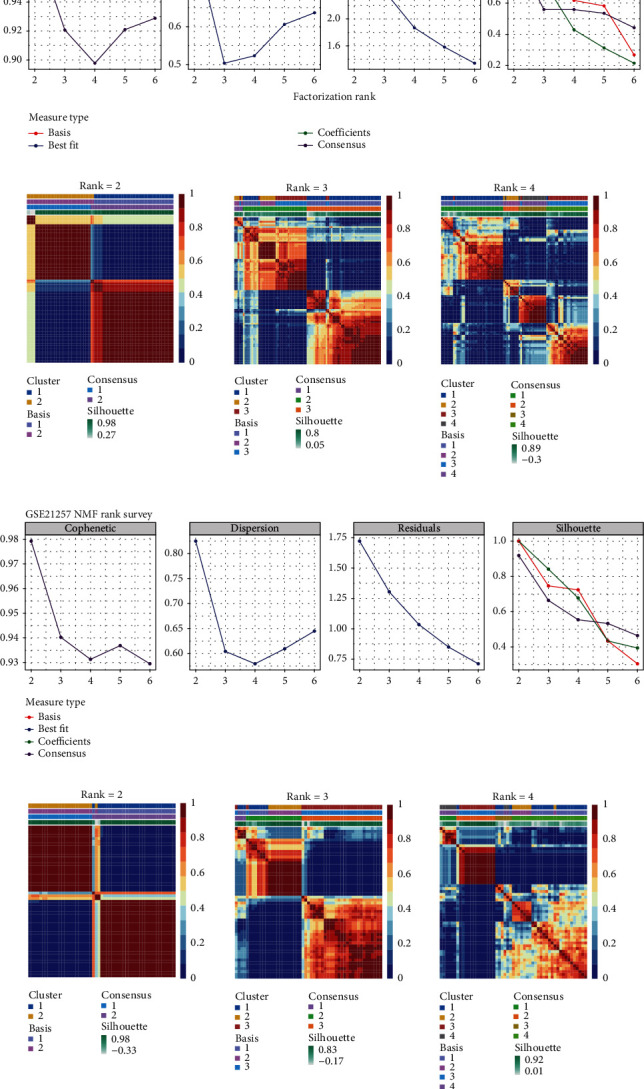
Identification of immune subtypes of osteosarcoma in training and validation cohort by NMF consensus clustering. The optimal clustering number was determined by the cophenetic, dispersion, residual, silhouette indicators, and clustering heatmap.

The study flowchart is shown in Figure 1. In this study, we found that an optimal clustering number was 2 in both cohorts based on cophenetic, dispersion, residual, silhouette indicators, and clustering heatmap in NMF analysis. Osteosarcoma samples were identified into two clusters which were low tumor immune infiltration cluster (L-TIIC) and high tumor immune infiltration cluster (H-TIIC) (TCGA: 46 L-TIIC (54.12%) vs. 39 H-TIIC (45.88%), 29 L-TIIC (54.72%) vs. 24 H-TIIC (45.28%)) (Figures 2(a)–2(d)).

### 3.2. L-TIIC Characterized by Poor Overall Survival

**Figure 3:**
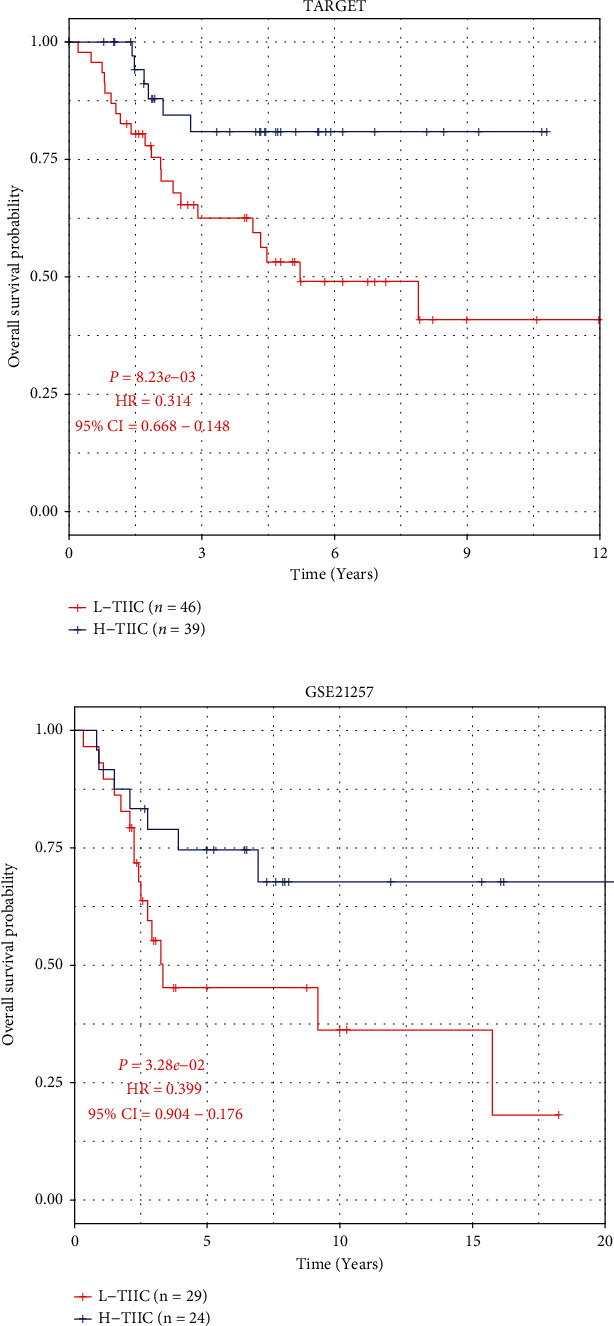
Overall survival of H-TIIC and L-TIIC osteosarcoma subtypes in (a) TARGET cohort and (b) GSE21257 cohort.

There were no significant differences in clinical characteristics between the two subtypes in either cohorts (Table 1). But there was a significant survival difference in survival analysis. L-TIIC had poorer overall survival than H-TIIC in TCGA cohort (*P* < 0.05, HR = 0.314, 95%CI = 0.148 − 0.668, Figure 3(a)). We also found similar results in the GSE21257 cohort (*P* < 0.05, HR = 0.399, 95%CI = 0.176 − 0.904, Figure 3(b)).

### 3.3. Differential Tumor Immune Microenvironment

**Figure 4:**
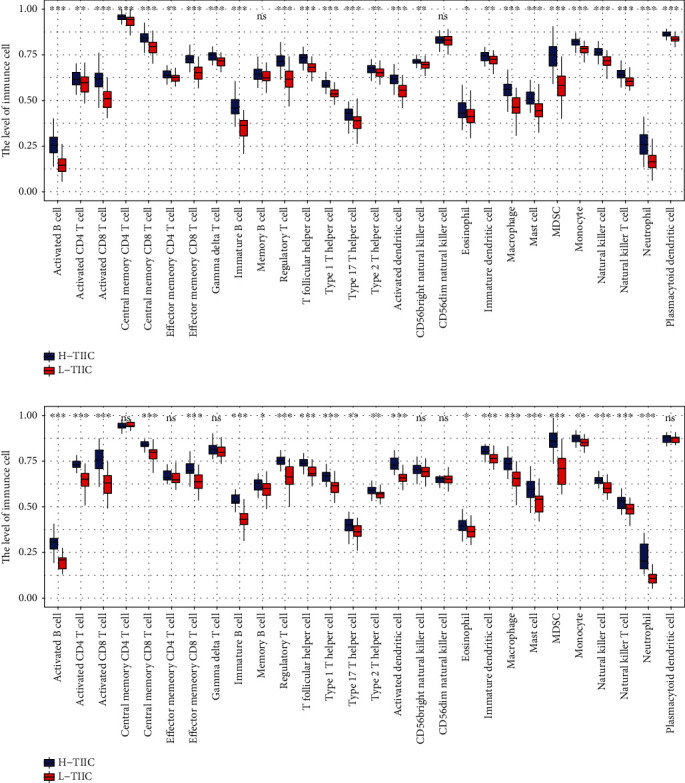
The infiltration of 28 immune cells between H-TIIC subtype and L-TIIC subtype in (a) TARGET cohort and (b) GSE21257 cohort.

In our analysis, we observed that many kinds of immune cells existed significant differences between two subtypes (Figure 4). It indicated that two subtypes had different immune status.

### 3.4. Gene Set Enrichment Analyses

**Figure 5:**
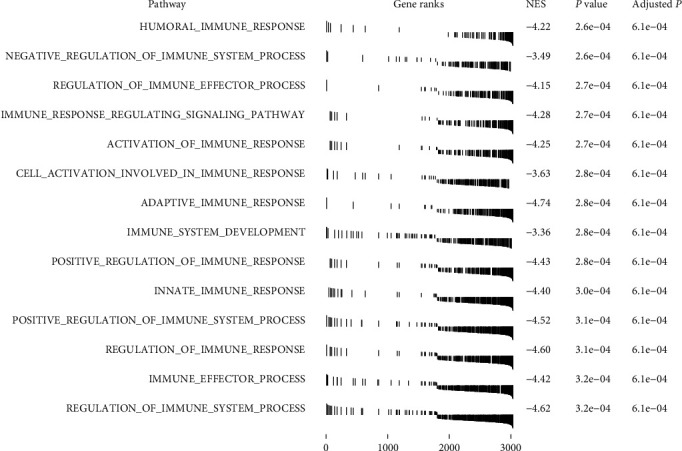
Differentially enriched signaling pathways between H-TIIC subtype and L-TIIC subtype.

In GSEA analysis, our results revealed that many biological processes differed between the two immune subtypes (Figure 5). Most of them were related with immune process. It indicated that two subtypes existed different immune responses, which may result in poor prognosis in L-TIIC.

### 3.5. Differential Expression of Immune Checkpoint Molecules and Immune Therapeutic Response

**Figure 6:**
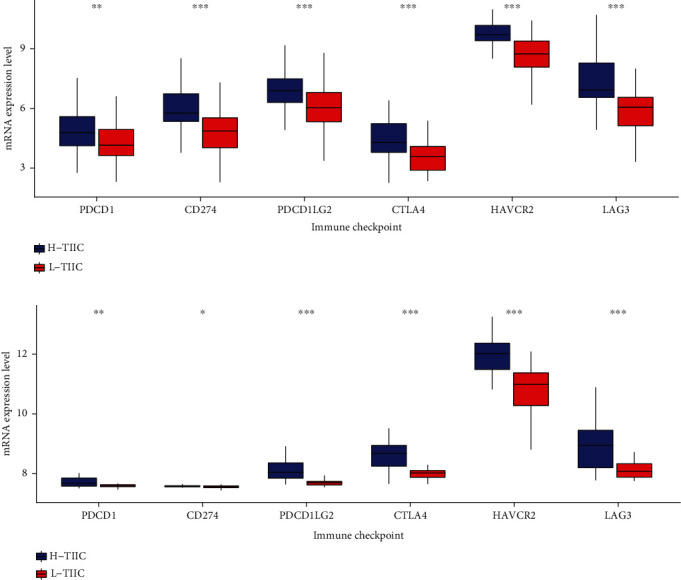
Expression of immune checkpoints between H-TIIC and L-TIIC subtypes in (a) TARGET cohort and (b) GSE21257 cohort.

**Figure 7:**
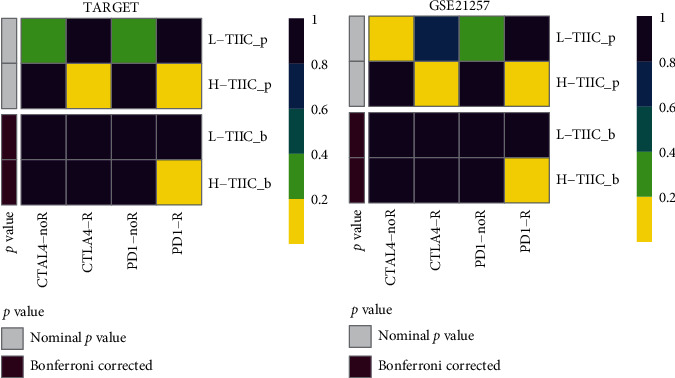
Submap analysis predicted the response to anti-PD-1 and anti-CTLA4 therapy for H-TIIC and L-TIIC subtypes in (a) TARGET cohort and (b) GSE21257 cohort. R represents immunotherapy responders. Bonferroni corrected *P* value < 0.05 was considered statistically significant.

Our analysis also found that expression of immune checkpoint molecules (PDCD1, CD274, PDCD1LG2, CTLA4, and HAVCR2) in L-TIIC was significantly lower than H-TIIC (Figure 6, *P* < 0.05). Moreover, immunotherapeutic response analysis indicated significant differences in anti-PD1 treatment responses (Figure 7).

### 3.6. Differences in Sensitivity of Chemotherapy

**Figure 8:**
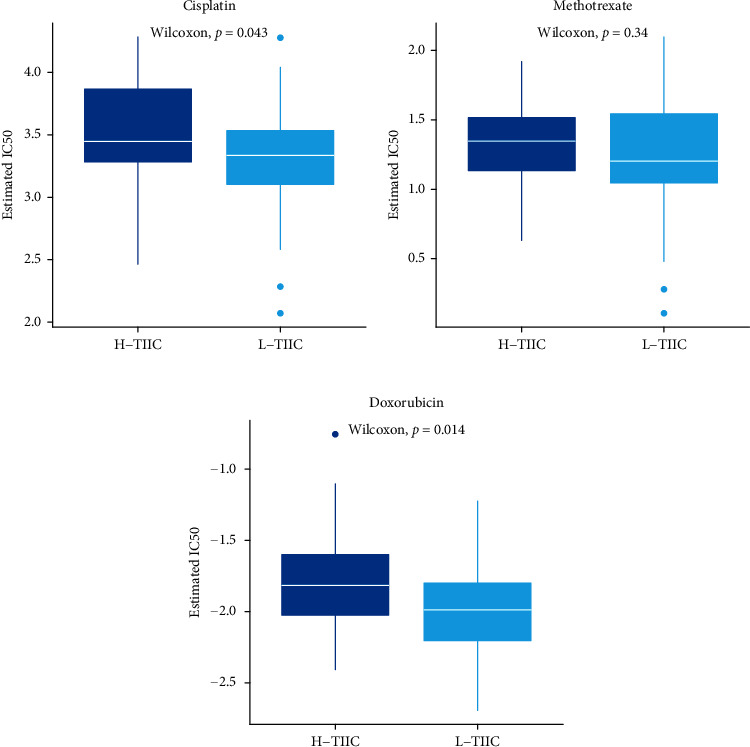
Chemotherapeutic sensitivity between high and low TIIC subtypes. Estimated median inhibition concentration (IC50) of (a) cisplatin, (b) methotrexate, and (c) doxorubicin.

In the chemotherapeutic drug sensitivity analysis, it declared that two chemotherapeutic drugs (cisplatin and doxorubicin) existed significant sensitivity differences between the two subtypes (*P* < 0.05, Figures 8(a)–8(c)).

## 4. Discussion

In the last decade, comprehensive treatment method has been a significant benefits for osteosarcoma [20]. However, a small part of patients still received a poor survival outcomes [21, 22]. With the in-depth recognition of TIME, the differential tumor microenvironment has been confirmed to play an important role in the poor survival outcomes of tumors [23, 24]. Therefore, the exploration of TIME is a chance for improving therapeutic effect and prognosis in osteosarcoma.

In this study, we successfully established two novel immune subtypes based on TIME of osteosarcoma. The results revealed that L-TIIC subtype is characterized by a poor prognosis. It could be speculated that the differences in tumor immune microenvironment resulted in this outcomes. Studies declared that TIME plays a complex role, such as promoting or inhibiting the proliferation of tumors [25, 26], promoting apoptosis of tumor cells and immune cells [27], increasing resistance to chemotherapy [28], and reducing or inhibiting ability of fighting tumor [29]. Many immune cells have obvious difference in two subtypes. B cells could produce antibodies and secrete cytokines to regulate antitumor immune process [30]. But it also reduces antitumor ability by inhibiting proliferation of immune-activated T cells [31]. CD8 T cell positivity is regulated by killing tumor cells [32]. Moreover, CD8 T cell is closely related with immunity therapy in osteosarcoma [33], and it is the result of differences in immunotherapy targeting PDCD1 (PD-1) in our analysis. Studies have revealed that mast cell, MDSC, macrophages, and monocyte could upregulate inhibitory receptors and produce immunosuppressive cytokines that are associated with poor prognosis in osteosarcoma [8, 34, 35]. The complex and differential immune environment could reduce differential immune processes. Our gene set enrichment analysis confirmed that there are a large number of different immune-related processes between the two subtypes.

Three chemotherapy regimens (methotrexate, adriamycin, and cisplatin) have been shown to be effective in many osteosarcoma patients [36]. But, there are still some patients who do not benefit from it because of developing drug resistance [22, 37]. In our analysis, we could observe that the sensitivity of the H-TIIC immune subtype to cisplatin and doxorubicin was lower than that of the L-TIIC subtype. We speculate that the H-TIIC has higher drug resistance. The cause of chemotherapy resistance is a complicated topic. *PD-1*, *CD274* (*PD-L1*), *PDCD1LG2* (*PD-L2*), *CTLA-4*, *HAVCR2* (*TIM3*), and *LAG-3* were expressed at higher levels in the H-TIIC subtype. Studies have uncovered that immunosuppressive cells in tumor environment could bind these inhibitory receptors to increase chemotherapy resistance [38–41]. In addition, studies have found that elimination of B lymphocytes could significantly increase the sensitivity of patients to chemotherapy [42, 43]. We believe that difference of TIME might lead to different sensitivity of cisplatin and doxorubicin. Our views are consistent with previous studies [22]. Therefore, exploration of TIME provided a new route in study of osteosarcoma.

This study achieved some good results and provided useful help for clinical decision in osteosarcoma. However, there still exist some drawbacks. For example, the relevant public data could not directly offer the immune cell composition ratio of each sample and lack of animal experiments and clinical cohort validation. Thus, larger data cohorts and more experiments are needed to verify classification and prognostic differences in clinical practice.

## 5. Conclusions

Taken together, we had successfully distinguished two immune subtypes, which could clearly reflect the heterogeneity of the immune microenvironment of different osteosarcomas. Two immune subtypes were characterized by differential overall survival, immune-related biological processes, sensitivity of chemotherapy, and response of ICB treatment. Among them, the established H-TIIC subtype has a good effect on the improvement of chemotherapy resistance of osteosarcoma. This study could provide clinical decision reference for treatment of osteosarcoma.

## Figures and Tables

**Table 1:** The relationship between two immune subtypes and clinical features.

Variable	GSE21257			TARGET		
	H-TIIC (*n* = 24)	L-TIIC (*n* = 29)	*P* value	H-TIIC (*n* = 39)	L-TIIC (*n* = 46)	*P* value
Age (median)	17.33	15.08	0.124	14.09	14.43	0.433
Gender			0.275			0.122
Female	11	8		21	16	
Male	13	21		18	30	
Metastasis (at diagnosis)			0.076			0.779
No	21	18		34	38	
Yes	3	11		5	8	
Huvos grades			0.462			
1	3	10				
2	8	8				
3	7	6				
4	3	2				
Unknown	3	3				

## Data Availability

The data used to support the findings of this study are included within the supplementary information files.

## Abbreviations

FDR: False discovery rate
ICB: Immune checkpoint blockade
NES: Normalized enrichment score
NMF: Nonnegative matrix factorization
ssGSEA: Single-sample gene set enrichment analysis
TARGET: Therapeutically Applicable Research to Generate Effective Treatments
TIIC: Tumor immune infiltration cluster
TIME: Tumor immune microenvironment.

## Supplementary Materials

Supplementary Materials See S1-28 immune gene set in the Supplementary Material for quantified TIME level of each sample with 28 immune signatures.
